# Assessing Birds of Prey as Biological Pest Control: A Comparative Study with Hunting Perches and Rodenticides on Rodent Activity and Crop Health

**DOI:** 10.3390/biology14091108

**Published:** 2025-08-22

**Authors:** Naama Ronen, Anna Brook, Motti Charter

**Affiliations:** 1 Shamir Research Institute, University of Haifa, Katzrin 1290000, Israel; 2 School of Environmental Sciences, Faculty of Social Sciences, University of Haifa, Mount Carmel, Haifa 3498838, Israel

**Keywords:** rodent control, barn owl (*Tyto* spp.), hunting perches, biological pest management, machine learning (YOLOv5), unmanned aerial systems (UAS), predatory pressure

## Abstract

Rodent damage significantly affects agriculture around the world. Rodenticides can sometimes control pests, but they are costly, may cause secondary poisoning to nontarget wildlife, and can become less efficient over time due to bait shyness and resistance. Using wildlife as biological pest control agents, particularly barn owls (*Tyto* spp.), has been suggested as an alternative. Barn owl nest boxes and hunting perches have been added to increase predator pressure, yet few studies have examined their effectiveness. We conducted a field study in forty-five 10 × 10 m^2^ plots to compare three treatments (biological pest control by adding hunting perches, 1080 rodenticide, and control) on rodent (vole) activity and crop health (alfalfa, *Medicago sativa*) using unmanned aerial system (UAS) remote sensing and ground surveys. Additionally, we used 24/7 video cameras and a machine learning (YOLOv5) object detection algorithm to determine whether hunting perches increase the presence of diurnal and nocturnal raptors. Rodent activity increased during the study and did not vary among the treatments across all three treatment groups, indicating that neither the biological pest control nor the rodenticides prevented the rodent population from increasing. Moreover, the vegetation indices clearly showed that the alfalfa has become increasingly damaged over time, due to the rising damage caused by rodents. There were significantly more raptors in plots with hunting perches than in control plots and those treated with rodenticides. Specifically, barn owls and diurnal raptors (mainly black-shouldered kites) spent 97.92% more time on hunting perch plots than rodenticide plots and 97.61% more time on hunting perch plots than control plots. The number of barn owls was positively related to vole activity, indicating a bottom-up process, while the number of black-shouldered kites was unrelated to vole activity. Even though hunting perches effectively increased the presence and activity of diurnal and nocturnal raptors, rodent populations increased. Future research should investigate whether hunting perches can increase raptor populations and improve crop health in crops beyond alfalfa, which is known to be particularly challenging to control for voles.

## Simple Summary

Rodents cause severe damage to crops worldwide, and while rodenticides are often used to control them. These poisons can harm other animals and may lose effectiveness over time. One natural solution is to use birds of prey, like barn owls, as biological pest control agents to hunt rodents. In this study, we tested whether adding wooden poles, called hunting perches, could attract more birds of prey and help protect alfalfa crops from rodents. We compared three treatments located in alfalfa fields: one with hunting perches, one treated with rodenticide poisons, and one left alone (control). We used drones, ground checks, and video cameras to track rodent activity and bird visits. Although more birds of prey—especially barn owls and black-shouldered kites—visited the plots with perches, rodent numbers still increased in all plots, and crop damage got worse over time. This suggests that while hunting perches attract helpful birds, they did not stop the rodents from increasing and damaging the alfalfa. Future studies should investigate whether this method is more effective in other crop types that are more susceptible to rodent damage.

## 1. Introduction

Meeting the rising demand for agricultural produce requires both agricultural expansion and intensified production, which pose severe threats to biodiversity [1]. Forty percent of the world’s land is used for agriculture, and pesticide use has serious effects on wildlife and nearby ecosystems [2,3,4,5]. Crop yield is influenced by a variety of factors, including weather, soil, topography, and agricultural practices such as fertilizers and irrigation [6,7,8]. Furthermore, crop yield is also affected by biotic factors, including pests (i.e., insects, snails, rodents) and diseases [9,10,11].

Controlling rodents is difficult because it is labor-intensive, expensive, and multiple treatments are needed to be effective. Rodenticides can be highly effective [12], but they sometimes lose their efficiency over time as rodents develop resistance and bait aversion [13]. Rodenticides can have detrimental effects through secondary poisoning of non-target wildlife species such as birds of prey, mammals, and amphibians [14,15,16]. 

The rise in recognition of ecosystem services [17], heightened concern over the impacts of chemical pesticides [18], and the recognition of the importance of sustainable agriculture for both biological conservation and human well-being has renewed interest in the role of natural enemies as agents of biological control. Specifically, natural enemies are being reconsidered as key agents in integrated pest management strategies [19]. Barn owls (*Tyto* spp.) are widely used in biological control projects of rodents in Israel [20,21], the USA [22,23,24,25,26,27], along with some other countries such as Greece, Cyprus, and Jordan [28]. In addition to barn owls, diurnal raptor populations, such as common kestrel (*Falco tinnunculus*), can also be increased in specific fields by adding nest boxes [29,30,31,32,33]. The use of barn owl nest boxes in Israel was first introduced in the Hula Valley in the 1960s and has since spread. Today, the use is widespread, with a positive outlook among most farmers [21]. The use of barn owl nesting boxes is considered cheaper than trapping at low rodent densities; however, the former is not as efficient as the latter at high rodent densities [34]. Additionally, it remains uncertain whether rodents are affected by the addition of barn owl nest boxes [35] and/or the presence of the owls in specific fields.

Raptors such as barn owls, common kestrels, and black-shouldered kites (*Elanus caeruleus*) use different techniques in search of prey, such as flight, hovering, and perching (also known as sitting and waiting). Predation success and hunting methods may be limited by the availability of hunting perches [29]. Even though raptors can hunt by flight or hovering over fields without perches, it is energetically more costly than from perches [36]. Although artificial hunting perches have been added to increase raptor presence [37,38,39,40,41,42,43,44,45], their effectiveness remains uncertain because most studies lack control plots and use monitoring camera devices with limitations. One of the difficulties is the methodology used in monitoring hunting perches, mainly using human observation and camera traps. Determining the presence of raptors using human observations [33,37,40,41,42,46] is limited mainly to diurnal birds of prey because of limited visibility at night, and the presence of humans may affect or even reduce raptor activity [47].

Camera traps are used [38,43,45,48] as an alternative to human observation. However, they were primarily designed to monitor large game mammals and rely on motion or heat triggers, which are often not sensitive enough to detect birds that are smaller and faster than mammals. Even in cases where the motion/heat trigger detects birds, the birds may not be filmed because most cameras’ triggers have a delay, so flying birds may be missed altogether and may no longer be near the camera. In addition to the limitations of the trigger mechanisms, most cameras either capture still images or videos of a specific length, making it impossible to determine the duration of perch use and overall raptor activity. Using 24/7 video surveillance cameras will provide continuous coverage both day and night.

The impact of adding hunting perches on rodent populations and crop yields remains unclear. A study in the Pacific Northwest, USA, found no significant effect of adding hunting perches on hunting pressure on the rodent population [37]. In another study, an experiment using an enclosure plot found that although raptors showed a clear preference for plots with perches, their presence did not affect the vole populations [41]. Kestrel visitation increased 11-fold in another study [42] in plots with artificial perches compared to control plots. Although the vole population size, growth rate, and adult survival were similar, juvenile recruitment was lower, and the proportion of reproductive females was reduced in plots with artificial perches compared to control plots. In the Czech Republic, a study based on human observations found that diurnal raptor density was higher in fields with artificial perches but did not find any effect on rodent abundance [49]. Lastly, the question of whether the increased presence of raptors affects crop yield and health remains critical. Although hunting perches were found to reduce mouse population growth rate and density compared to control plots in a study conducted in Australia, crop damage remained similar between treatments [40]. There is a need for studies that monitor artificial perch use in conjunction with 24/7 video cameras, rodent activity, and crop yield and health.

In addition to assessing raptor presence and rodent activity, it is crucial to evaluate vegetation health to track rodent damage. Small unmanned aerial systems (UAS) have recently been utilized in precision agriculture and wildlife surveys [50]. UAS has been employed to assess crop biomass and yield [51], as well as pest damage [52,53]. Additionally, remote sensing data from UAS can be utilized to identify rodent damage [54,55] and rodent burrow detection [56]. Using UAS, studies have found negative relationships between rodent populations and crop health, and positive relationships between the number of burrows and rodents [54,57].

We studied whether installing hunting perches as a form of biological rodent control affects rodent activity and crop health, compared to rodenticide-treated and untreated control plots. We hypothesized that both hunting perches and rodenticides would reduce rodent activity and improve vegetation health relative to the control plots. The second goal was to determine if hunting perches could increase the presence of diurnal and nocturnal raptors. We hypothesized that hunting perches would significantly increase both the presence and duration of stay for diurnal and nocturnal raptors.

## 2. Materials and Methods

### 2.1. Study Area

The study occurred in four alfalfa (*Medicago sativa*) fields (mean = 29.34 ha, range = 17.4- 34.7 ha, Standard Error = 4.13 ha) in the Hula Valley area located in the northeast of Israel (33°6′15.7′′ N, 35°36′26.1′′ E) during the late fall until the early spring (November 2021–April 2022). During this period, farmers do not harvest the alfalfa, allowing us to work freely without disturbing agricultural activities. The study area has a large barn owl population with many nest boxes monitored over the years. Between 2012 and 2022, the average number of barn owl pairs was 51 (standard error = 3.1, range = 36–68 pairs) [58]. We focused our study on the vole population increase period from fall to spring to determine when barn owls can effectively control vole growth. During this time, owls begin courtship, lay eggs, and the first nestlings hatch. Peak hunting activity happens when the oldest barn owl nestlings are 20–25 days old, as both parents hunt together, which coincides with the peak in vole numbers. However, most crop damage has already occurred by then. This suggests that effective vole control methods should focus on reducing population growth earlier in the season, before peak vole numbers and crop damage occur.

Alfalfa is a perennial crop mainly used for animal feed, is highly nutrient-dense, and is, therefore, one of the most valuable forage crops [59,60]. In Israel, Günther’s vole (*Microtus guentheri*) is a common rodent pest [54], causing significant damage in various agricultural areas. Although alfalfa fields are typically cultivated for up to five years, they are often abandoned after just 2–3 years due to severe damage caused by voles.

### 2.2. Experimental Design

Forty-five, 10 × 10 m plots were established for each of the three treatments (15 plots per treatment): biological pest control (Hunting perches), rodenticides (1080), and controls. After the plots were selected (see below), they were marked by four 0.75 m poles bordered with strings. Each field had an equal number of treatments, and the mean number of plots was 11.25 (standard error = 2.25). Each plot was further divided into five 10 × 2 m strips, delineated by ribbons at the plot’s edges, used while counting rodent burrows. Plots were 100 m to the nearest neighboring plot, which is sufficient to reduce raptor visibility between plots due to the vegetation [36] and ensure that plot spacing exceeds vole movement ranges [61].

#### The Three Treatments Were

**Figure 1:**
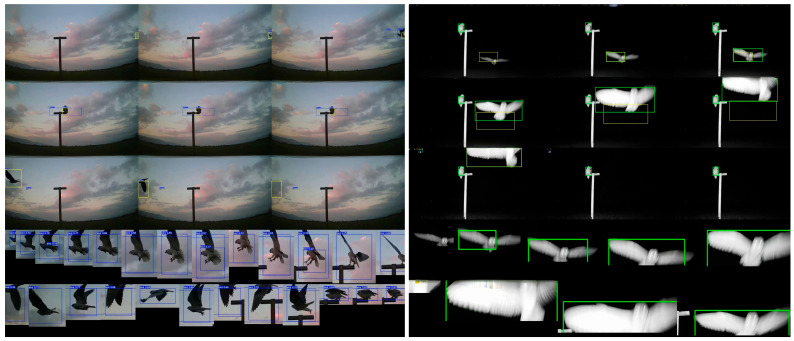
Examples of screenshot images from video of the object tracking (Section 2.5.2) of a perching black-shouldered kite during the day (**left**) and a barn owl flying with another owl in the background (**right**). The numbers around the boundary boxes represent the confidence score and are not essential to understanding this figure.

1.  Hunting Perches plots had one T-shaped wooden perch (2.2 m tall, 0.4 m long crossbar) that was positioned at a distance of 5.65 m from the south side of the plot, allowing the raptors a place to stand (Figure 1). There were also no natural or other manmade structures that raptors could use for perches within the areas. The perch height was selected based on findings indicating that raptor visibility is minimal to almost nonexistent at distances of 100 m when the vegetation height is 7 cm [36].
2.  1080 (Rodenticide) plots received a treatment of 30 g of 0.05% Sodium fluoroacetate (mixed into a wheat bait) (known as Rosh80 or 1080). 1080 rodenticide was uniformly dispersed across the plots during two applications. The first application was made after data collection in session 1, and 2nd application was added ten days later. 1080 is the only rodenticide legally permitted for use in open agriculture in Israel and has been used with the same bait for 28 years [62]. It is highly toxic to wildlife and humans, and no known antidote exists [62,63].
3.  Control plots had no treatment applied.

### 2.3. Site Selection and Pre-Experimental Evaluation

#### 2.3.1. Imaging of Fields

The goal was to assess pre-existing differences in the vegetation index and rodent activity. The four fields were imaged using a DJI Phantom 4 Pro ((DJI, Shenzhen, China), using Pix4Dcapture (version 4.11.0) software at an altitude of 75 m (GSD of approximately 1.94 cm/px), 80% overlap, and a camera angle of 80° during November 2021. Five-eight ground control points (GCPs) were marked in each field, and coordinates were measured using a Zenith 15 & 25 Pro Series GNSS (GeoMax AG, Widnau, Switzerland) with a accuracy of 4 cm accuracy. White calibration panels were placed on the ground to normalize the pixel values. Spectral data was collected using an OceanOptics USB4000 350–1100 nm spectrometer (Ocean Optics, Inc., Dunedin, FL, USA), with a spectral resolution of 0.5 nm, and OceanView 1.6.7 software was used to calibrate the UAS data. The spectrometer underwent calibration using a white calibration panel.

#### 2.3.2. Pre-Processing

The images, including five to eight GCPs, were processed using the structure from motion (SfM) method, in Pix4D Mapper Pro (v. 4.3.31), producing orthophoto, DSM, and 3D models.

#### 2.3.3. Calculating Vegetation Indices

The orthophoto results were normalized using the white calibration boards’ pixel’ values and their spectral signature collected [54]. The orthophotos were masked to include only vegetation using a biomass index (green-blue/2-red/2), where each pixel in a normal RGB image receives a value for the biomass index ranging from 0 to 70 mg biomass per gram of dry matter and a threshold to get the plant cover [64]. These results were then oversampled to 18 wavelengths and calibrated using the empirical line (EL) method and the spectral signature collected from the alfalfa crop [65]. Lastly, three vegetation indices (NDVI, PRI, and SIPI) were calculated. Additionally, the vegetation cover percentage was calculated using the mask mentioned above and implemented with ENVI Classic 5.1 software, and then further processed with Python 3. PRI and SIPI are used as an index of vegetation health monitoring (NV5 Geospatial Solutions, Exelis Visual Information Solutions, 20152025)

#### 2.3.4. Rodent Burrow Count

Previous studies have found that the number of rodent burrows is related to the number of voles that were trapped and can be used as an index of vole activity [54,66]. Rodent activity was measured through ground surveys performed by a single trained field worker. All visible rodent burrow entrances within each plot were manually identified and counted. To estimate activity, we used the burrow renewal rate method: each visible burrow was covered, and after two days, the number of reopened burrows was recorded [54]. This method assumes that burrows reopened within this timeframe reflect active rodent presence and behavior. Therefore, the burrow renewal rate provides a non-invasive and cost-effective proxy for estimating rodent abundance and spatial activity in the field.

#### 2.3.5. Selection of Potential Plots and Statistical Analysis

No significant pre-existing differences existed in the number of rodent burrows and NDVI among the 45 plots of the three treatment types (Table 1).

### 2.4. Data Collection

All 45 experimental plots (15 plots per treatment type) were measured using (1) an Unmanned Aerial System (UAS) for imaging to assess vegetation indices, (2) Rodent burrow surveys to quantify rodent activity, and (3) 24/7 video cameras to monitor both nocturnal and diurnal raptor presence and perch utilization during three sessions: (session 1) mid-January to early February, (session 2) mid-February to early March, and (session 3) mid-March to early April.

#### 2.4.1. Imaging of Plots

Before conducting burrow surveys, UAS imaging was performed to avoid any disturbance to the alfalfa caused by field personnel. The same imaging protocol described in Section 2.3.1 was used, including the DJI Phantom 4 Pro drone and Pix4Dcapture software (version 4.11.0). The resulting orthophotos were processed and calibrated using the previously described methods, and vegetation indices (Table 2) were calculated to assess crop health during each sampling session.

#### 2.4.2. Rodent Burrow Count and Alfalfa Cover Estimation

The renewal rate of rodent burrows was determined (see Section 2.3.4). Also, the percentage of alfalfa cover was calculated as the proportion of alfalfa relative to the total vegetation (alfalfa plus weeds). This was visually estimated by a single trained observer who conducted the assessment within each 10 × 10 m plot, immediately following the rodent burrow surveys. The same observer performed all estimations to ensure consistency across plots and sessions. This visual assessment was carried out during the first two sampling sessions to provide a standardized measure of vegetation composition, which could influence rodent activity and habitat use. Due to logistical constraints, including limited time and field accessibility, this measurement was not conducted during the third session.

#### 2.4.3. Video Cameras

We added a surveillance solar camera system consisting of two 12V 22AH/20HR batteries housed in a plastic box with a stand, a 1 m metal pole, a 1.2 m wooden pole, a solar charge controller (SRNE Solar CO., LTD, Shenzhen, China), a 30 W solar panel (Zhejiang ERA Solar Technology Co., Ltd., Taizhou, China) (35 × 65 cm), an Exir Mini Bullet surveillance camera (Model: DS-2CD2021G1 4 mm B, Hikvision, Hangzhou, China), a 128 GB Sandisk Ultra memory card, and a flexible multicore cable (3182Y, H05VV-F, unscreened, 2 core, 1.5 mm^2^) [70]. The camera setup was added at an 8 m distance from the plots’ south side and set to view the edge of the fields in 8 control plots, 13 hunting perch plots, and 11 rodenticide plots. Pigeon spikes and solar panels were added to the camera to prevent birds from using the camera as a perch. RGB footage was captured during the day, while infrared (IR) imaging was recorded at night for 48 h across two of the three data collection sessions (18 in session 2 and 14 in session 3).

### 2.5. Processing Video Camera Footage

**Figure 2:**
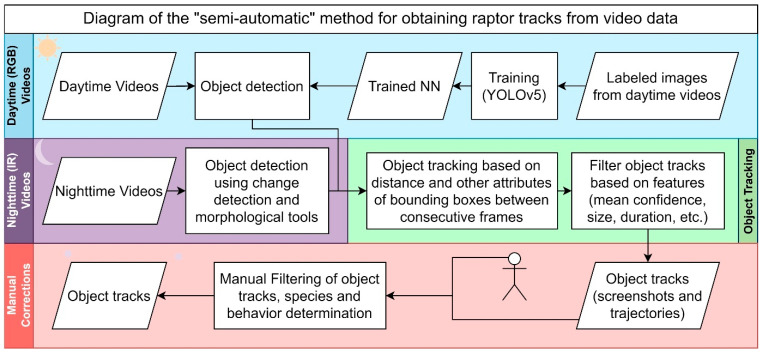
Flowchart of the Semi-Automatic Method for Raptor Track Extraction from Video.

Video footage from field cameras was analyzed to determine the presence and time spent by diurnal and nocturnal raptors. The raptors’ behavior was categorized as flying (the act of moving through the air using wings), hovering (remaining stationary in the air by flapping wings quickly), or perching (hunting, resting, or sitting on an elevated surface). Manual assessment of videos (viewing the full videos) is very time-consuming, so we developed a “semi-automatic” method to analyze the videos. Object detection algorithms were first applied to detect birds in each frame of the video. Then, the objects in consecutive frames were re-identified to become a track/event, which was automatically then manually filtered, with the behavior and species determined (Figure 2).

#### 2.5.1. Step 1: Detection of Birds per Frame

Analysis of daytime videos (RGB) was conducted using machine learning with the YOLOV5 network, which was pre-trained on the MS-COCO dataset and trained with 850 bird-labeled images from videos of randomly selected plots and dates, and validated with 500 images. The resulting model, with a recall of 0.7 and a precision of 0.5, was used to detect birds in videos by displaying a bounding box with its confidence level. Nighttime videos were captured using IR sensors for which we had no available pre-trained network. Since there was less noise at night, bird objects were detected using change detection and morphological image processing tools.

#### 2.5.2. Step 2: Object Tracking

After each frame, bird object predictions were estimated, and object tracking was performed based on the distance between bounding boxes in consecutive frames, size differences, and trajectory. Several blank frames in the tracks were tolerated to prevent false negatives from the previous step from splitting the tracks due to the low recall. The resulting object tracks were then filtered based on mean bounding box size, average confidence level, track duration, and the number of frames with the object.

**Figure 3:**
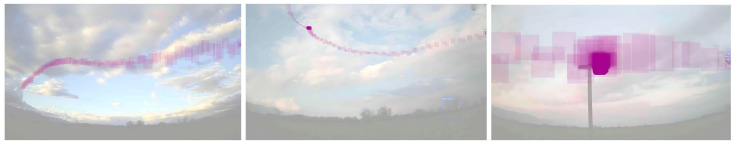
Trajectory images showing raptors during the day flying (**left**), hovering (**center**), and perching (**right**).

For each of the filtered tracks, the system generated a table with event attributes (plot information, start and end times) and two other output files: 1. Composite Frame Analysis File (Figure 1): Contains nine images arranged as follows: First three frames of the detected event, middle three frames of the detected event, and last three frames of the detected event. This includes two rows of extra screenshots showing the start and finish of the detected event. 2. Trajectory Visualization File (Figure 3) demonstrates objects’ complete trajectory by overlapping their bounding boxes’ positions in each event frame, using the first frame as the background.

#### 2.5.3. Step 3: Manual Review

**Figure 4:**
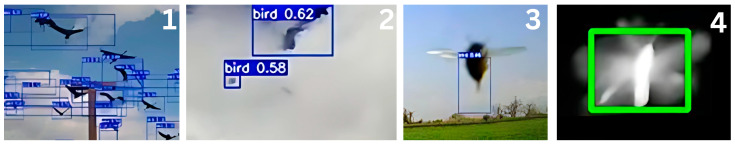
False positive examples: (**1**) a flock of Eurasian cranes (*Grus grus*), (**2**) clouds, and (**3**,**4**) insects falsely classified as birds. The numbers around the boundary boxes represent the confidence score and are not essential to understanding this figure.

The two output files were reviewed to detect false positives and non-raptor species removal (see examples in Figure 4), identify raptor species, and classify the raptors’ behavior.

#### 2.5.4. Step 4 Validation

To evaluate the accuracy of the semi-automatic raptor detection system using camera footage, we compared its outputs to manual annotations (Table 3). Each detection event was classified as a True Positive (TP; a raptor was correctly detected), False Positive (FP; a raptor was incorrectly detected), or False Negative (FN; a raptor was missed). Based on these classifications, we calculated two performance metrics: Recall (TP/[TP + FN]), which measures the system’s ability to detect actual raptor events, and Precision (TP/[TP + FP]), which reflects the proportion of correct detections among all positive identifications. This semi-automatic method was validated on 20 h of daytime videos and 22.5 h of nighttime videos, sampled from randomly selected plots in alfalfa fields across various dates, encompassing a total of 88 raptor detection events.

### 2.6. Statistical Analysis

All tests were performed using IBM SPSS Statistics 23 software. We used the Kruskal–Wallis chi-square test to assess whether there were any pre-existing differences in the number of rodent burrows and NDVI values among the different treatment groups at the start of the experiment. We used a Generalized Linear Mixed Model (GLMM) with Poisson (for count data) and Normal (for continuous and negatively skewed variables). Gamma distributions (for continuous and positively skewed variables) and log-linked/identity functions to determine whether the number of burrows counted and vegetation indices measured varied across sections and in the treatment types, with field as a random variable to avoid pseudoreplication. We initially included the interaction between treatment and session in the GLMM. However, the interactions were not statistically significant (*p* > 0.05) and were therefore removed from the final model to improve interpretability and model parsimony. We used pairwise contrasts with the least significant difference (LSD) to test for differences between treatment types. We used a similar model to determine whether the number of events caught on the cameras varied in the treatment types and whether it was related to the number of rodent burrows. Model diagnostics were performed, including checks for overdispersion and visual inspection of residuals to assess model fit. These steps revealed no significant violations of model assumptions.

## 3. Results

### 3.1. Treatments’ Effect on Vole Activity and Alfalfa Health

**Figure 5:**
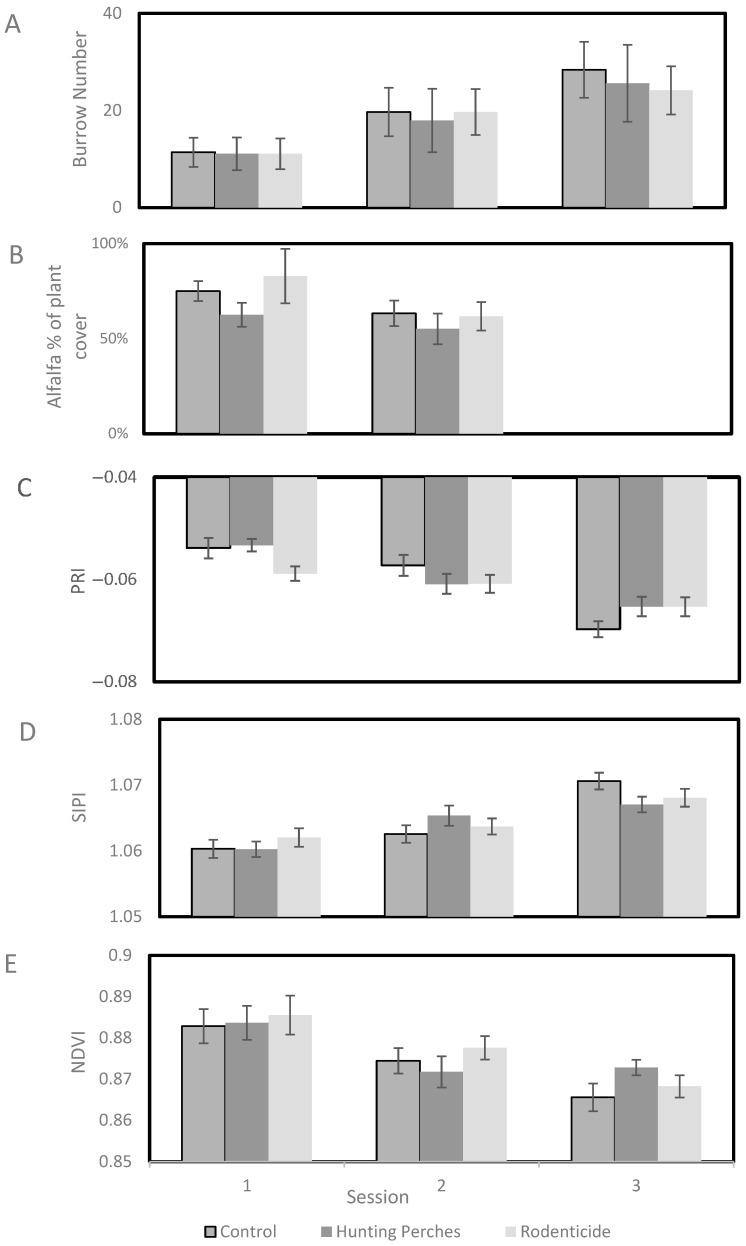
The burrow numbers (**A**), percentage of alfalfa cover (**B**), PRI (**C**), SIPI (**D**), and NDVI (**E**) values for the three treatments (Hunting Perches, *n* = 13; Rodenticide, *n* = 11; and Control, *n* = 11) during the three data collection sessions (1: mid-January to early February, 2: mid-February to early March, 3: mid-March to early April).

We used a Generalized Linear Mixed Model (GLMM) with a Poisson distribution and a log link function, including field as a random effect, for the following analyses. In a GLMM (F_4,130_ = 64.41, *p* < 0.001) to compare the three treatments (hunting perch, rodenticide, and control plots) and the three sessions, there was significant variability in the number of burrows between the sessions (F_2,130_ = 126.82, *p* < 0.001) (Figure 5A), with an increase in rodent burrows over the season. In contrast, there was no significant difference in the number of rodent burrows between the treatments (F_2,130_ = 2.0, *p* = 0.14).

Using a GLMM with a Gamma distribution and a log link function (field as a random effect), the percentage of alfalfa plant cover varied between the first and second sessions and treatments (F_3,83_ = 4.56, *p* = 0.005). Specifically, the percentage of alfalfa plant cover showed significant differences between the sessions (F_2,83_ = 4.14, *p* < 0.05), indicating a decrease over the seasons (Figure 5B). Likewise, there was a difference between the treatments (F_1,83_ = 5.28, *p* < 0.05), with marginally but not significantly more alfalfa plant cover observed in the perch plots compared to the control (*p* = 0.058) and rodenticide plots (*p* = 0.075).

Using a GLMM with a normal distribution and an identity link function (field as a random effect), the PRI index was analyzed during the three sessions and treatments (F_4,115_ = 10.39, *p* < 0.001). Specifically, the PRI showed significant differences between the sessions (F_2,115_ = 19.70, *p* < 0.001), indicating a decrease over the seasons (Figure 5C). However, there was no significant difference in the PRI between the treatments (F_2,115_ = 1.01, *p* = 0.34).

Using a GLMM with a Gamma distribution and a log link function (field as a random effect), the SIPI index was analyzed in the three sessions and treatments (F_4,115_ = 10.34, *p* < 0.001). Specifically, the SIPI showed significant differences between the sessions (F_2,115_ = 20.60, *p* < 0.001), indicating an increase over the seasons (Figure 5D), meaning a decrease in vegetation health. However, there was no significant difference in the SIPI between the treatments (F_2,115_ = 0.08, *p* = 0.92).

Using a GLMM with a Gamma distribution and a log link function (field as a random effect), the NDVI was analyzed during the three sessions and treatments (F_4,115_ = 10.37, *p* < 0.001). Specifically, the NDVI showed significant differences between the sessions (F_2,115_ = 20.07, *p* < 0.001), indicating a decrease over the seasons (Figure 5E). However, there was no significant difference in the NDVI between the treatments (F_2,115_ = 0.07, *p* = 0.55).

### 3.2. Perch Use

The 13 cameras in hunting perch plots recorded 318 events involving raptors using the perches over 624 h (mean = 24.5 events, SE = 4.6 events, 0.51 events per hour), with raptors using the perches for 25.3 h of the video (mean = 1.9 h, SE = 0.4 h).

### 3.3. Comparison of the Number of Perching Events Between the Treatments

Using a GLMM with Poison distribution and log-linked function and field as a random variable (F_2,28_ = 32.55, *p* < 0.001), the number of times raptors were present varied across the treatments (F_2,28_ = 33.34, *p* < 0.001) and was positively related to the number of rodent burrows (F_1,28_ = 13.24, *p* = 0.001). Specifically, using a pairwise contrast with the least significant difference (LSD) adjusted significance level, the number of raptors that used plots with hunting perches was greater than the number in the rodenticide plots (*p* < 0.001) and the control plots (*p* < 0.001). The number of raptor events for hunting perch plots was 54.5% of the total number of events, the rodenticide plots were 27.7%, and the control was 17.8%.

**Figure 6:**
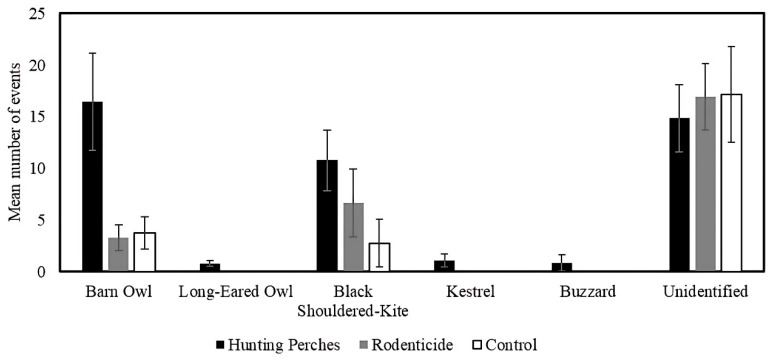
The mean number of events by different raptor species appearing on cameras in the three treatments: Hunting Perches (black, *n* = 13), Rodenticide (gray, *n* = 11), Control (white, *n* = 11), over 48 h.

Using a similar GLMM (F_3,28_ = 46.82, *p* < 0.001), we found that the number of barn owls varied between treatments (F_2,28_ = 47.43, *p* < 0.001) (Figure 6) and was positively related to the number of rodent burrows (F_1,28_ = 18.49, *p* < 0.001). Specifically, using a pairwise contrasts with the least significant difference (LSD) adjusted significance level, the number barn owls that used plots with hunting perches (mean = 16.5 events, SE = 4.7) was more than in the rodenticide (mean = 3.8 events, SE = 1.6) (*p* < 0.001) and control plots (mean = 3.3 events, SE = 0.1) (*p* < 0.001) (Figure 6).

In comparison, using a similar GLMM (F_3,28_ = 13.54, *p* < 0.001), the number of black-shoulder kite varied between the treatments (F_2,28_ = 19.99, *p* < 0.001) (Figure 6) but was not related to the number of rodent burrows (F_1,28_ = 2.43, *p* = 0.13). Using pairwise contrasts with the least significant difference (LSD) adjusted significance level, the number of black-shouldered kites only marginally significantly differed among the hunting perch, rodenticides, and control plot types (*p* > 0.05 < 0.10) (Figure 6).

### 3.4. Comparison of the Time Spent Between the Treatments

**Figure 7:**
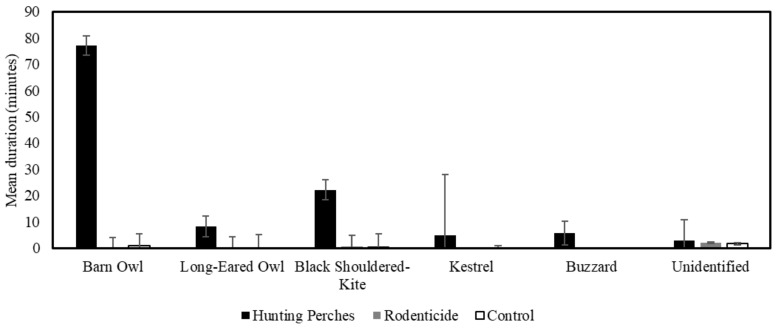
The mean duration (minutes) of different raptor species spent in the three treatments, Hunting Perches (black, n = 13), Rodenticide (gray, n = 11), Control (white, n = 11), in 48 h.

Overall, raptors spent 97.92% more time on hunting perch plots than on rodenticide plots and 97.61% more time on hunting perch plots than on control plots. Specifically, barn owls spent 99.93% more time on hunting perch plots than on rodenticide plots, and they spent 98.81% more time on hunting perch plots than on control plots. Black-shouldered kites spent 97.61% more time on hunting perch plots than on rodenticide plots, and 98.29% more time on hunting perch plots than on control plots (Figure 7).

Using a GLMM with Gamma distribution and log-linked function and field as a random variable (F_3,27_ = 68.42, *p* < 0.001), the duration spent (minutes) by the raptors were present during treatments varied (F_2,28_ = 98.57, *p* < 0.001, Table 1) but was not related to the number of rodent burrows (F_1,28_ = 2.05, *p* = 0.16). Using a pairwise contrasts with the LSD adjusted significance level, the duration spent (minutes) in plots with hunting perches (mean = 121.1 min, SE = 26.1) was more than the rodenticide (mean = 2.6 min, SE = 0.5) (*p* < 0.001) and control plots (mean = 3.0 min, SE = 1.2) (*p* < 0.001).

In a similar GLMM (F_3,24_ = 54.8, *p* < 0.001), the duration spent by barn owls varied between treatments (F_2,24_ = 78.6, *p* < 0.001, Figure 7) but was only marginally related to the number of rodent burrows (F_1,24_ = 3.8, *p* = 0.06). Lastly, using a GLMM (F_3,19_ = 13.2, *p* < 0.001), the duration spent by black-shouldered kites varied during treatments (F_2,24_ = 19.6, *p* < 0.001, Figure 7) but was not related to the number of rodent burrows (F_1,19_ = 1.6, *p* = 0.22).

### 3.5. Comparison Between Hunting Strategies in the Treatment Plots

Using a GLMM with field as a random variable, the number of raptor events perching varied across the three treatments (F_2,29_ = 22.5, *p* < 0.001) (Table 4). Specifically, using pairwise contrast with the LSD, the number of raptor events perching in the plots with hunting perch was higher than in the rodenticide (*p* < 0.001) and control plots (*p* < 0.001). In another GLMM (F_2,29_ = 0.37, *p* = 0.70), the number of raptor events hovering did not vary between treatments. Similarly, the number of raptor events flying did not vary between treatments (F_2,29_ = 1.11, *p* = 0.34).

Using GLMM, the duration spent by raptors perching also varied during treatments (F_2,29_ = 16.4, *p* < 0.001). Specifically, using pairwise contrasts with the LSD significance level, the duration spent by raptors perched in the hunting perch plots was higher than in the rodenticide (*p* < 0.001) and control plots (*p* < 0.001). In another GLMM, with field as a random variable (F_2,29_ = 0.60, *p* = 0.57), the duration spent by raptors hovering did not vary between treatments. Similarly, the duration spent by raptors flying also did not vary between treatments (F_2,29_ = 0.40, *p* = 0.68).

### 3.6. The Relationship Between Rodent Burrows and the Duration Raptors Spent

**Figure 8:**
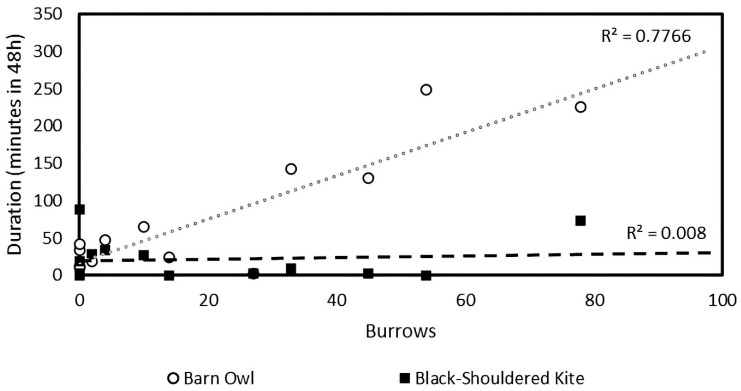
The relationship between the number of burrows counted and duration spent by barn owls (open circle trendline, *n* = 13) and Black-Shouldered Kite (dashed black line, *n* = 13) in Hunting Perches- treated plots.

The duration spent by barn owls increased with the number of rodent burrows in the hunting perch plots (F_1,11_ = 21.72, *p* = 0.001, Figure 8), but was not in the rodenticide (F_1,7_ = 0.36, *p* = 0.57) and control plots (F_1,4_ = 218.0, *p* < 0.001). The duration spent by black-shouldered kites was not related to the number of rodent burrows in the hunting perch (F_1,11_ = 0.08, *p* = 0.78, Figure 8), rodenticide plots (F_1,5_ = 1.87, *p* = 0.23), or control plots (F_1,11_ = 2.89, *p* = 0.34).

## 4. Discussion

### 4.1. Treatment Effects on Raptor Activity and Vole Activity in Alfalfa Fields

This study aimed to investigate whether natural predators (biological control agents) can have a similar effect on rodents as rodenticides in alfalfa fields. Surprisingly, the number of vole burrows was not only similar across the treatments but also increased over the course of the study in all three treatments. The rise in vole numbers shows that both natural predators and rodenticides failed to control the vole population completely. In addition to the increase in rodent burrows, the vegetation indices (PRI, SIPI, and NDVI) all indicate that the alfalfa has become more damaged over time. Other studies have also found that vegetation indices are inversely related to both rodent numbers and burrows [54,57]. The increased damage indicates that the treatments were ineffective and are closely linked to rising rodent activity.

One reason we may not have observed any changes in rodent numbers is that we selected alfalfa fields, which are known to be problematic for rodent control because alfalfa is high in protein, rich in minerals, and easily digestible [71], and may be preferred over bait such as the wheat bait with 1080 used in this study. Voles are recognized explicitly for causing significant damage to Alfalfa fields [54,57]. Furthermore, the rodenticide 1080 was used in Israel for 29 years with the same bait (wheat) [62,72,73], and there is a possibility that voles have developed bait-shyness. If the voles do not eat the bait, then rodenticides are useless. Furthermore, alfalfa is a perennial plant that is harvested multiple times a year by trimming; it is not plowed, thereby preserving vole burrows [21]. In-depth and extensive research efforts to test different rodenticides and baits are necessary to determine which are more effective and efficient, just as has been performed in the USA [74,75,76,77].

Predators may be unable to control the vole population because voles do not need to forage far from their burrows in alfalfa fields. Barn owls are recognized as specialists in hunting small mammals, primarily preying on voles [78,79,80]. They also reproduce more when the vole population is high [81]. Likewise, black-shouldered kites are known as a vole specialist [82]. It is therefore highly likely that both barn owls and black-shouldered kites preyed on voles during the study period. Vole populations are characterized by high fecundity rates, where individuals can produce multiple litters within a single breeding season [83,84]. There is a high likelihood that the voles could reproduce rapidly, which may allow them to produce more offspring than the raptors can remove in crops such as alfalfa.

The study was conducted during the early breeding phase of barn owls (courtship, egg incubation, and newly hatched nestlings) when vole abundance and crop damage are minimal. The idea is to prevent vole populations from reaching extremely large numbers while the vole numbers are still small. Although raptor hunting activity increases later in the season as adults provision larger nestlings, this coincides with peak vole abundance and significant crop damage, which reduces the potential for effective intervention. Nevertheless, future studies conducted later in the season could provide valuable insights into whether perch use increases and whether raptor activity can eventually stabilize or reduce vole populations.

There is also the possibility that the hunting perches influenced not only the plots in which they were installed but also adjacent plots, including control plots. Ideally, we would have increased the distance between plots to minimize this potential spillover effect; however, this was not feasible due to the limited size of agricultural fields in Israel compared to other countries. We do not consider this to be a limitation because the perches were 2.2 m high and at least 100 m away from the nearest neighboring plot, a distance previously shown to decrease raptor visibility in tall vegetation such as alfalfa [36]. While the raptors most likely did not hunt far due to a lack of visibility from the perches [36], there is the possibility that the raptors would use the perches to rest and hunt by flight in the other plots. Regardless, neither treatment decreased rodent activity nor reduced damage observed in the vegetation indexes.

It is possible that without the treatments, the vole population may have increased more; however, this is unlikely given that both rodent burrow and vegetation indices were similar between the two treatment plots and the control plots. There is a need to determine whether including hunting perches and 1080 rodenticides may be effective in crops other than alfalfa.

Additionally, we recognize that the relatively short duration of the trial, limited to a single growing season, may have restricted our ability to detect long-term ecological trends or population-level effects. While our findings offer valuable insights into short-term dynamics, longer monitoring over multiple seasons would be needed to evaluate cumulative impacts on both raptor and rodent populations. However, conducting such long-term studies involves significant technical and logistical challenges, including maintaining solar-powered surveillance systems, managing data storage and processing for continuous video footage, and ensuring ongoing access to agricultural fields. Despite these challenges, we acknowledge the importance of long-term research and hope future studies can address these complexities.

### 4.2. The Use of Hunting Perches

Hunting perches significantly increased the number of visits and the presence of both diurnal and nocturnal raptors compared to the rodenticide and control plots. Two main small mammal specialists utilized hunting perches: the barn owl at night and the black-shouldered kite during the day. We found that the duration that barn owls used the perches was related to vole burrow numbers and activity. In contrast, no such relationship was found in black-shouldered kites, which may be attributed to the voles’ being primarily nocturnal [85]. Previous studies have shown that artificial perches caused an increase in raptor abundance in the treated plot areas during the day only [37,40,41,42]. In these studies, data collection regarding perch utilization primarily relied on visual surveys, which are limited to the daytime, and the presence of observers during these surveys can potentially influence raptor behavior [47]. Other studies have utilized camera traps that primarily determine presence or absence. However, due to the sensitivity of the triggers, many birds may not be captured on film [38,39,43,45]. Using 24/7 continuous video recordings allowed us to calculate not only presence and absence during both day and night, but also to determine the duration of time perches were used.

An interesting aspect of our study was that we monitored not only perches but also plots without any perches (control and rodenticide plots). This setup allowed us to determine whether the perches increased the presence of diurnal and nocturnal raptors. The plots without perches helped us assess whether there was an increase in the raptors’ presence and behaviors. Here, we found that the number and duration of raptors flying and hovering did not vary between the plots with perches and those without. Although we placed perches at least 100 m from neighboring plots to minimize overlap, this distance was based on prior findings suggesting that raptor visual detection and hunting efficiency decrease significantly beyond this range in dense vegetation such as alfalfa [36]. However, we acknowledge that some raptors may still hunt beyond this distance, particularly by flight or hovering, which could have influenced adjacent plots.

Future studies should aim to not only investigate whether birds of prey use hunting perches, but also integrate tracking devices such as GPS [86,87] or reverse GPS [88,89] to determine how exactly the perches enhance hunting efficiency and practices. Hunting perches can potentially be used not only to increase predation but also could affect the behavior/distribution of prey population by the “ecology of fear” [90,91] in crop types other than alfalfa.

We found that all the perches were utilized, which was consistent with other studies that used camera traps: one conducted in Indiana reported that 82% were used [45], while another in California indicated that 83.3% were utilized [38]. In comparison, when examining perch events per total hours, we found 0.51 in this study compared to much lower figures in Indiana (0.003) [45], California (0.012) [38], and another study in California (0.02) [43]. Since each study collects and analyzes data following unique protocols, comparisons were not always possible. For example, some studies provide the number of photographs taken rather than unique events [39].

We found that perches were used for 4.1% of the total time, which is relatively high. Uniquely, our study quantified not only the frequency but also the duration of these behavioral events. Although camera traps have been used to estimate event durations [43], their design for short, motion-triggered clips with reset delays makes them unsuitable for monitoring long-duration behaviors. The high usage of perches in this study is likely attributable to habitat variation, as the alfalfa fields examined likely support higher rodent densities compared to the habitats in previous studies. Although the perch height used in our study (2.2 m) was lower than in previous studies [38,43], raptor activity was higher, suggesting that the use of taller perches might have led to even greater utilization, although this still needs to be studied.

The higher number of events per hour and the more extended use of the perches may be due to having more raptors in the alfalfa fields compared to the other studies. Still, it may also result from the 24/7 continuous recording cameras. In the other studies [38,43,45,48], some events may have been missed entirely by observers or trail cameras, as mentioned above, or the increased events found in this study may be due to the detection method. Using 24/7 continuous recording cameras has many advantages compared to trail cameras for monitoring birds. Continuous video surveillance provides a more accurate representation of raptor presence, as the typical motion tracking triggers used in commercial trail cameras work poorly, especially for fast-flying small animals such as birds. Furthermore, it is impossible to accurately calculate the duration of perching and identify and analyze behaviors such as flying and hovering using trail cams. There are also disadvantages. Recording 24/7 requires a much bigger system, including a camera, battery, and solar panel (the costs are roughly the same). This setup requires more time to install and dismantle and frequent data card replacements since they fill up quickly and need ample storage. The primary drawback is that analyzing the camera data requires coding knowledge, which can vary across locations. We developed a custom algorithm that was more time-efficient than manually reviewing entire videos; however, it is complex and may not be accessible to individuals without coding knowledge. Although the cost of a solar-powered system is similar to that of trail cams, the latter is labor-intensive. As a result, using too many cameras at once is impossible due to the difficulties in setting them up and switching storage cards.

### 4.3. Numerical and Functional Response

The functional response describes how the predation rate changes with prey density, and the numerical response is defined as the change in the number of prey through immigration and reproduction as prey density varies [92,93,94]. In our study, although hunting perches significantly increased both diurnal and nocturnal raptor presence and activity, the vole population continued to grow. The short-term numerical response was not enough to offset the high reproductive rate of voles in the alfalfa fields.

Artificial perches may not only facilitate hunting [36] but also enhance breeding success by increasing the foraging efficiency of adult raptors. In barn owls, higher hunting success results in more prey being brought, which is positively linked to clutch size and brood size [81,95]. Over time, this could result in more breeding pairs and a higher number of nestlings, especially in landscapes where nest boxes are already available and well-used, as is the case in our study area.

This potential for population-level growth in raptors could amplify predation pressure in subsequent seasons, creating a delayed but stronger numerical response. Such a response may be particularly important in perennial crops like alfalfa, where rodent populations persist year-round and require sustained control efforts. Moreover, the presence of hunting perches may encourage territorial settlement by additional raptor pairs, especially in areas where natural perching or nesting structures are limited.

However, this long-term benefit is contingent on several factors: the availability of suitable nesting sites [58], minimal disturbance during the breeding season, and sufficient prey availability to support larger broods. In our study region, where barn owl nest boxes are abundant and monitored, the addition of perches could synergistically support both adult survival and reproductive output, potentially leading to a cumulative increase in predator density over time.

## 5. Conclusions

This study demonstrates that while artificial hunting perches significantly increased the presence and activity of both diurnal and nocturnal raptors (mainly barn owls and black-shouldered kites), the raptors and rodenticides were insufficient to reduce vole activity or crop damage in alfalfa fields effectively. Rodent populations increased across all treatments, including those with rodenticides and perches, indicating that neither method alone was sufficient to suppress vole growth in this crop system.

The findings highlight the limitations of biological control in problematic perennial crops, such as small alfalfa fields, where dense vegetation and high rodent reproduction may reduce predator efficiency. The high usage of perches by raptors may enhance predator foraging opportunities and may contribute to long-term increases in raptor populations through improved hunting success and reproductive output. The integration of 24/7 video surveillance and machine learning (YOLOv5) provided a novel and practical approach for quantifying raptor behavior and perch use, offering a valuable tool for future ecological monitoring.

We recommend that future research explore the effectiveness of biological pest control using hunting perches in other crop types. Additionally, studies should assess whether combining perches with other pest management strategies, such as alternative rodenticides, can yield more effective and sustainable outcomes. Long-term monitoring of different crop types for crop damage and perch uses across seasons will be essential to fully evaluate the potential of hunting perches as a scalable tool in integrated pest management.

## Figures and Tables

**Table 1:** Preexisting Similarities Among Selected Plots: The 15 plots chosen for the three treatments did not differ significantly in rodent activity (burrow counts) or vegetation index (NDVI) at the start of the study.

Treatment	Burrows		NDVI	
	Mean	Std Dev	Mean	Std Dev
Hunting Perches (*n* = 15)	11.1	12.6	0.884	0.017
Rodenticide (*n* = 15)	11.1	11.8	0.886	0.017
Control (*n* = 15)	11.4	17.0	0.883	0.015
Kruskal–Wallis	H = 0.54, df = 2, *p* = 0.76		H = 0.15, df = 2, *p* = 0.93	

**Table 2:** Description of the three vegetation indices used in this study.

Vegetation Index	Equation	Description	Source
NDVI (Normalized Difference Vegetation Index)	$\frac{R_{800}-R_{680}}{R_{800}+R_{680}}$	Measures the presence and health of vegetation in an area.	[67]
PRI (Photochemical Reflectance Index)	$\frac{R_{531}-R_{570}}{R_{531}+R_{570}}$	Measures sensitivity to changes in carotenoid pigments in live foliage, which are indicative of photosynthetic efficiency. Drops indicate increased canopy stress.	[68]
SIPI (Structure Insensitive Pigment Index)	$\frac{R_{800}-R_{445}}{R_{800}-R_{680}}$	Measures leaf pigment concentrations normalized for variations in overall canopy structure and foliage content. Increases in SIPI indicate increased canopy stress.	[69]

**Table 3:** Events and performance metrics for the semi-automatic analysis. Evaluation of camera identification was calculated as True Positives (TP, a raptor was correctly detected), False Positives (FP, a raptor was wrongfully detected), False Negatives (FN, a raptor was not detected), Recall ((TP/(TP + FN)), and Precision (TP/(TP + FP)).

Time	Behavior	TP	FP	FN	Recall	Precision
Day	Flying	11	4	2	0.85	0.73
	Hovering	14	2	2	0.88	0.88
	Perching	8	0	0	1.00	1.00
Night	Flying	3	1	0	1.00	0.75
	Perching	41	0	0	1.00	1.00

**Table 4:** Mean number of raptor visits and time spent (minutes) in 48 h ± SE by different behaviors.

Number of Visits	Perching	Hovering	Flying
Hunting Perches (n = 13)	24.46 ± 4.64	2.69 ± 1.74	17.54 ± 4.12
Rodenticide (n = 11)	0.00 ± 0.17	1.64 ± 0.59	24.82 ± 4.92
Control (n = 8)	0.00 ± 0.17	1.13 ± 0.72	21.75 ± 5.23
Duration			
Hunting Perches (n = 13)	116.96 ± 25.97	0.99 ± 0.67	2.93 ± 1.05
Rodenticide (n = 11)	0.00 ± 0.17	0.44 ± 0.17	2.19 ± 0.41
Control (n = 8)	0.00 ± 0.17	0.28 ± 0.21	2.67 ± 1.04
